# HIV status is linked to higher odds of persistent respiratory symptoms in Tanzania: a longitudinal analysis

**DOI:** 10.5588/ijtldopen.25.0438

**Published:** 2025-12-10

**Authors:** J.S. Kreniske, G. Ruselu, S. Zhang, B. Desderius, S. Kalluvya, R.J. Kaner, D.W. Fitzgerald, R.N. Peck

**Affiliations:** 1 Division of Pulmonary and Critical Care Medicine, Weill Cornell Medicine, New York, NY, USA;; 2 Center for Global Health, Weill Cornell Medicine, New York, NY, USA;; 3 Department of Medicine, Catholic University of Health and Allied Sciences, Mwanza, Tanzania;; 4 Department of Medicine, New York-Presbyterian/Weill Cornell Medical Center, New York, NY, USA;; 5 Department of Genetics, Weill Cornell Medicine, New York, NY, USA.

**Keywords:** tuberculosis, chronic respiratory disease, smoking, sub-Saharan Africa

Dear Editor,

Even in the era of anti-retroviral therapy (ART), HIV is linked to persistent respiratory symptoms in high-income countries where tobacco smoking is common.^1,2^ However, the majority of people with HIV (PWH) live in sub-Saharan Africa (SSA) where tobacco smoking is less common, and where cross-sectional studies evidence conflicting results.^2–10^ Longitudinal data from SSA concerning HIV and persistent respiratory symptoms in the era of ART are lacking.^1^ We hypothesise that PWH are at elevated risk for persistent respiratory symptoms in a low-smoking SSA population with access to ART. We evaluated this hypothesis within a comparative cohort of people with and without HIV in Mwanza, Tanzania.

This was a longitudinal analysis of the ongoing Mwanza HIV&CVD study.^11^ A convenience sample of participants were recruited during their first HIV care visit at Bugando Medical Centre (BMC). PWH were ART-naïve, aged 18–65, Tanzanian citizens, and able to provide informed consent. Friends or family were recruited as controls. Exclusion criteria included history of cardiovascular disease (CVD), clinically evident heart failure, prognosis less than 12 months, or plans to move out of the Mwanza area. HIV testing was done at enrolment. Study visits (month 0, month 3, month 6, month 12, month 24) were completed between 2015 and 2020. Participants completed an adapted WHO STEPS questionnaire.^12^ CD4^+^ T-cell count was measured using an automated BD FACS Calibur machine and repeat HIV testing was performed at the 24-month visit for the control group. A standardised questionnaire assessed two respiratory symptoms at each visit: dyspnoea on exertion and disruptive cough (described as cough that disturbed the patient during rest). The primary study outcome was the presence of ‘persistent symptoms’, defined by at least one respiratory symptom reported at all visits (month 0, month 3, month 6, month 12, and month 24). Clinical diagnoses of respiratory infections and TB during the follow-up period were participant-reported and confirmed in the medical record. Data were analysed using Stata 18. The primary analysis was the association between HIV status and persistent respiratory symptoms. Covariates included in the multivariable model were identified a priori, based on established drivers of persistent respiratory symptoms. Ethical approval was obtained from the institutional review boards of Weill Cornell Medicine, the Tanzanian National Institute for Medical Research, and BMC. Informed consent was obtained in Kiswahili.

We enrolled 1,000 participants: 495 PWH and 505 controls. Fifty participants died prior to 24-month follow-up (47 PWH and 3 HIV-uninfected). Two hundred forty-four participants missed at least one follow-up visit and were excluded from the analysis. The 706 participants who completed all study visits were included in the analysis (307 PWH and 399 HIV-uninfected). All PWH initiated ART at baseline. Among the 706 included participants, the median age was 37 years (IQR 30–43 years) and 70% were female. Median CD4 count in PWH was 452 cells/mm^3^ (IQR: 203–620 cells/mm^3^), with 24% having CD4 < 200 cells/mm^3^ at baseline. Sixty-six percent reported monthly income below the international poverty standard of $1.90/day (66% of PWH and 66% of HIV-uninfected). Daily cigarette smoking was reported by 4% of PWH and 6% of HIV-uninfected participants, with a median of 0.25 packs smoked per day among smokers. Eight percent of PWH and 4% of HIV-uninfected participants were former smokers. Homeplace exposure to biomass fumes was reported by 83% of participants (88% vs. 79%). Workplace exposure to biomass fumes was reported by 16% of participants (17% vs. 13%). During a 24-month follow-up, 5 PWH and 7 HIV-uninfected participants were diagnosed with respiratory infections. New TB diagnosis during follow-up was reported by 5 PWH and 1 HIV-uninfected participant.

Persistent respiratory symptoms were identified in 20% of participants overall (141/706), including 25% (77/307) of PWH and 16% (64/399) of HIV-uninfected participants. PWH had higher odds of persistent respiratory symptoms in the first 2 years after ART initiation compared to HIV-uninfected controls followed up for the same time-period (OR: 1.75 [95% CI: 1.21–2.54], *P* value = 0.003). After adjusting for potential confounders, HIV status was associated with persistent respiratory symptoms in the multivariate model (aOR: 1.59 [95% CI: 1.07–2.36], *P* value = 0.020; see Table). Other risk factors independently associated with persistent respiratory symptoms in the multivariate model included respiratory infections, female sex, exposure to biomass fumes in the home, and income below the international poverty threshold.

**Table.** HIV status is linked to higher odds of persistent respiratory symptoms.

Variable	Odds ratio	95% CI	*P* value
People with HIV	1.59	(1.07–2.36)	0.020
Respiratory infection during follow-up	6.18	(1.73–22.12)	0.005
Female sex	2.70	(1.46–4.99)	0.002
Exposure to biomass fumes in the home	2.05	(1.05–4.00)	0.037
Income <$1.90/day	1.56	(1.01–2.42)	0.047
TB diagnosis during follow-up	3.95	(0.73–21.56)	0.112
Tobacco smoker (current)	1.99	(0.71–5.56)	0.190
Occupational exposure to biomass fumes	1.71	(0.97–3.02)	0.064
Tobacco smoker (former)	1.45	(0.56–3.74)	0.439
Age >50 years	1.28	(0.72–2.25)	0.400

In multivariate regression, HIV status is associated with persistent respiratory symptoms over 24 months of follow-up from ART initiation.

Among PWH, a history of treatment for TB prior to the study period was reported by 13% of participants overall (40/304), with an equivalent distribution among participants with and without persistent respiratory symptoms. Opportunistic infections during or prior to the study period were reported by only 2 participants, of whom 1 experienced persistent respiratory symptoms. Of PWH with nadir CD4 cell count less than 200 cells/mm^3^, 15% (11/75) reported persistent respiratory symptoms versus 29% (66/232) of those with nadir CD4 cell count greater than 200 cells/mm^3^. In this longitudinal study of PWH followed up for 2 years from the time of ART initiation, we found that HIV infection is associated with an increased risk of persistent respiratory symptoms in a low-smoking SSA setting. This finding corroborates observations from high-income/high-smoking burden populations and suggests that PWH are at elevated risk for cough and dyspnoea independent of tobacco exposure.^1^

Strengths of our study include a large cohort with 2 years of longitudinal follow-up at regular intervals from the time of ART initiation, detailed socio-demographic data and smoking history, and multivariable adjustment. Further, the present cohort underwent contemporaneous evaluation with echocardiography. Established CVD or clinically evident heart failure was an exclusion criterion, and the low rates of echocardiographic dysfunction have been previously published.^13^ Limitations of our study included the reliance on self-report for symptoms and exposure history. Further, respiratory symptom questions regarding cough and dyspnoea were formulated as a general screen and not derived from a comprehensive respiratory questionnaire.

The high overall prevalence of respiratory symptoms among people with and without HIV may reflect high levels of biomass smoke exposure or unmeasured exposures such as respiratory dysfunction due to TB prior to the study period. Female sex emerged as a strong predictor of respiratory symptoms, consistent with prior evidence of higher risk of respiratory symptoms and hypoxaemia among women in East Africa.^4,7,14^

Our findings speak to the urgent need to better understand and address non-smoking contributors to respiratory morbidity in SSA and the global south. Persistent respiratory symptoms are common among both PWH and HIV-uninfected adults in Tanzania but are more common in PWH. Other risk factors for persistent respiratory symptoms include female sex, respiratory infections, poverty, and homeplace exposure to biomass fumes. Comprehensive longitudinal investigation of respiratory symptoms and objective measures of lung function are needed in the epidemiologic contexts most relevant to PWH.
